# Geographic Access to Cancer Care and Treatment and Outcomes of Early-Stage Non–Small Cell Lung Cancer

**DOI:** 10.1001/jamanetworkopen.2025.1061

**Published:** 2025-03-18

**Authors:** Pratibha Shrestha, Ying Liu, James Struthers, Benjamin Kozower, Min Lian

**Affiliations:** 1 Division of Public Health Sciences, Department of Surgery, Washington University School of Medicine, St Louis, Missouri; 2 Alvin J. Siteman Cancer Center, Washington University School of Medicine, St Louis, Missouri; 3 Division of General Medicine & Geriatrics, Department of Medicine, Washington University School of Medicine, St Louis, Missouri; 4 Division of Cardiothoracic Surgery, Department of Surgery, Washington University School of Medicine, St Louis, Missouri

## Abstract

**Importance:**

Data on the impact of geographic access to cancer care on early-stage non–small cell lung cancer (NSCLC) treatment and outcomes are limited.

**Objective:**

To examine the associations of geographic access to cancer care with guideline-recommended treatment and outcomes in patients with early-stage NSCLC.

**Design, Setting, and Participants:**

This population-based cohort study included patients with early-stage NSCLC newly diagnosed between January 1, 2007, and December 31, 2015, followed up through December 31, 2016, and identified from the Surveillance, Epidemiology, and End Results dataset. Data analysis was performed from March to November 2024.

**Exposures:**

Geographic access to thoracic surgeons and radiation oncologists was quantified using the 2-step floating catchment area algorithm and categorized into quintile 1 (least access) through quintile 5 (greatest access).

**Main Outcomes and Measures:**

Multilevel logistic regression was performed to estimate odds ratios (ORs) of receipt of surgery and radiotherapy. Hazard ratios (HRs) of lung cancer-specific mortality were estimated using Fine and Gray subdistribution hazard regression.

**Results:**

Among 65 259 patients, the mean (SD) age was 69.4 (10.1) years; 33 114 patients (50.7%) were female, 1071 (1.6%) were uninsured, and 7541 (11.6%) were enrolled in Medicaid. The least (vs greatest) geographic access to thoracic surgeons (HR, 1.10; 95% CI, 1.03-1.18; *P* < .001 for trend) and radiation oncologists (HR, 1.11; 95% CI, 1.04-1.18; *P* < .001 for trend) was associated with higher lung cancer mortality. Patients in counties with the least (vs greatest) access to thoracic surgeons were less likely to undergo surgery (OR, 0.80; 95% CI, 0.69-0.93; *P* < .001 for trend); this association was much stronger in Asian than non-Hispanic White patients and in Medicaid-insured than non-Medicaid-insured patients. Although there was no significant association overall, geographic access to radiation oncologists was significantly associated with radiotherapy use in older (OR, 0.85; 95% CI, 0.76-0.95), Hispanic (OR, 0.65; 95% CI, 0.49-0.86), and uninsured (OR, 0.63; 95% CI, 0.43-0.94) patients.

**Conclusions and Relevance:**

In this cohort study, geographic access to cancer care was associated with guideline-recommended treatment for early-stage NSCLC and outcomes, particularly in socially marginalized patients, underscoring the importance of ensuring appropriate geographic allocations of cancer care resources and addressing travel barriers to health care to improve NSCLC treatment, prognosis, and equity.

## Key Points

**Question:**

Is geographic access to cancer care associated with guideline-recommended treatment for early-stage non–small cell lung cancer (NSCLC) and outcomes?

**Findings:**

In a population-based cohort study of 65 259 patients with early-stage NSCLC, those living in counties with the least (vs greatest) access to thoracic surgeons and radiation oncologists were more likely to die. The association between geographic access to lung cancer care and guideline-concordant treatment was stronger in older, Hispanic, Asian, uninsured, and Medicaid-insured patients.

**Meaning:**

These findings suggest that interventions targeting geographic barriers to lung cancer care could improve NSCLC treatment, prognosis, and equity.

## Teaser

This cohort study of patients with early-stage non–small cell lung cancer examines the association between geographic access to lung cancer care and patient outcomes.

## Introduction

Non–small cell lung cancer (NSCLC) comprises more than 80% of lung cancers.^1^ Improvements in lung cancer screening and treatment increased 5-year NSCLC survival from 18% in cases diagnosed between 1990 and 1992 to 26% in cases diagnosed between 2010 and 2016.^2,3^ Patients with early-stage tumors, including clinical stages I and II, represent 20% to 40% of NSCLC cases and have a 5-year survival rate of greater than 60%.^3,4^ The standard treatment for early-stage NSCLC is surgery. Stereotactic body radiation therapy (SBRT) is an alternative treatment option for patients with inoperable early-stage NSCLC.^5^ Despite treatment benefits, nearly one-quarter of patients remain undertreated.^1,6^ Sociodemographic factors have been associated with underuse of treatment for early-stage NSCLC, including older age, male sex, lower income, lack of insurance, Medicaid coverage, Black race, Asian race, Hispanic ethnicity, and residence in socioeconomically disadvantaged neighborhoods.^6,7,8^

Cancer treatment is a highly complex process and requires frequent visits to cancer specialists, indicating a critical role of geographic access to cancer care in high-quality treatment. Geographic access to health care is commonly measured using availability of nearby physicians and/or travel distance or time to the nearest treatment facility. Travel burden to health care has been reported as a barrier to screening, early detection, and treatment for some types of cancer.^9^ Alternatively, greater availability of physicians has been associated with early cancer detection, treatment, and better survival.^10,11,12,13^ However, little evidence for the association of geographic access to cancer care with lung cancer treatment is available. Studies have found that a travel distance of more than 100 miles to an operative facility was associated with lower odds of receiving stage-appropriate adjuvant therapy and postoperative surveillance imaging and higher risk of death among patients with early-stage NSCLC.^14,15,16^ By contrast, Obrochta et al^6^ reported that longer travel time to treatment facilities was associated with decreased risks of undertreatment and delays in initial treatment for early-stage NSCLC in California. Among Medicaid beneficiaries with lung cancer, neither travel distance nor travel time to primary care physicians was associated with late-stage lung cancer diagnosis and time to treatment.^17^ The discrepancy in findings is possibly due to the differences in patient characteristics and metrics used to quantify geographic access to care. These studies are limited by the inclusion of patients only from a single hospital, Commission on Cancer–accredited hospitals, or a single state. Compared with Commission on Cancer–approved hospitals, nonapproved hospitals are more likely to be smaller and located in rural areas and less likely to provide oncologic services.^18^

To overcome these limitations, we quantified geographic access to cancer care using a robust geospatial measure integrating travel burden to and availability of nearby thoracic surgeons and radiation oncologists and examined its association with early-stage NSCLC treatment and outcomes. We also assessed this association in patients who generally experience lung cancer treatment and outcome disparities, as geographic accessibility and availability of health care are potential drivers of cancer health disparities.^19^

## Methods

### Patient Population

We used the Surveillance, Epidemiology, and End Results (SEER) Research Plus dataset, which contains data from 18 registries (released in April 2019), to identify men and women with clinical stages I and II NSCLC (*International Classification of Diseases for Oncology*, *3rd Edition* [*ICD-O-3*] histological codes^1^) as a first primary malignant tumor diagnosed at 20 years or older between January 1, 2007, and December 31, 2015, and followed up through December 31, 2016 (n = 65 997). Patients were excluded if they were identified through autopsy or death certificate (n = 92) or had no information on residential counties (n = 82) or surgery or radiotherapy (n = 564). The final sample included 65 259 patients. Deidentified data were used, exempting the study from review and informed consent by the Washington University School of Medicine institutional review board. We followed the Strengthening the Reporting of Observational Studies in Epidemiology (STROBE) reporting guidelines.^20^

### Geographic Access to Cancer Care

Availability of physicians (determined using the ratio of physicians per number of persons within a geographic area) and travel distance or time to the nearest services are commonly used to define geographic access to health care. However, the former does not account for cross-border movements, and the latter ignores the competition among service facilities. The 2-step floating catchment area (2SFCA) algorithm overcomes these limitations by integrating available health sources and population demand and accounting for distance decay and service competitions.^21,22^ We demonstrated that 2SFCA outperformed service density and travel distance or time in the assessment of geographic access to mammography facilities.^21^ Because modeling of real street-based network distances between residential counties and service locations across a large geographic area requires high-quality street network data and results in a prohibitively high computational burden, we refined the 2SFCA algorithm using the euclidean distance to capture catchment areas. We quantified geographic access to thoracic surgeons and radiation oncologists separately by using the refined 2SFCA.

We used the National Plan and Provider Enumeration Systemic (NPPES) database, developed and regularly updated by the Centers for Medicare & Medicaid Services, to obtain primary or secondary practice locations of physicians with primary or secondary specialty as thoracic surgery and radiation oncology (or therapeutic radiation) who provided health care from 2007 to 2016. Practice locations of thoracic surgeons and radiation oncologists were geocoded to identify their corresponding census tracts, using ArcGIS, version 10.6.1 (ESRI). A catchment area of a specialist was defined based on straight-line distances of 30, 40, and 50 miles for metropolitan, urban, and rural areas, respectively, because travel patterns vary greatly among the 3 areas. The 2010 US Census data were used to obtain the population size in each census tract. We computed the distance matrix between each physician and population-weighted centroids of census tracts within a catchment area. The physician to population ratio for each physician was estimated by dividing the number of physicians by the weighed population of all counties where centroids fell into the catchment area of that physician. An access score for each census tract was calculated by summating the weighted physician to population ratios. We weighed the population and physician to population ratio using a gaussian 6-zonal slow-decay weighting function with a greater weight (more accessible) for population-physician pairs having shorter driving distances. The resulting geographic access index integrated travel burden to and availability of thoracic surgeons and radiation oncologists in surrounding areas. On the basis of geographic distributions of populations within a county, we aggregated the access scores from the census tract to county level. Quintiles of geographic access scores were created based on their distributions in all patients, with quintile 1 indicating the least access.

### Dependent Variables

Surgical treatment was determined using SEER lung surgery codes 12 to 90, including local tumor destruction, sublobar resection, lobectomy, pneumonectomy, and surgery not otherwise specified. Due to the lack of SEER codes specific for SBRT, patients treated with external beam radiation were considered to receive radiotherapy. We used the SEER cause-specific death classification variable to identify deaths attributable to lung cancer. SEER calculated follow-up months from the date of diagnosis to the date of death, date of last contact, or December 31, 2016, whichever occurred first.

### Covariates

Sociodemographic factors included age (20-49, 50-59, 60-69, 70-79, and ≥80 years), race and ethnicity (Asian, Hispanic, non-Hispanic Black, non-Hispanic White, and other [American Indian or Alaska Native, unknown, or any other race]), sex (male and female), health insurance (non-Medicaid insurance, Medicaid, no insurance, and missing), socioeconomic deprivation, and nonmetropolitan residence. Due to racial and ethnic differences in lung cancer treatment and outcomes,^23,24^ race and ethnicity were included as a covariate. SEER defined original racial and ethnic categories. A composite socioeconomic deprivation index was calculated for each county based on 21 variables from the 2008 to 2012 American Community Surveys^25,26^ and categorized into quintiles, with a higher quintile representing greater socioeconomic deprivation. Nonmetropolitan counties were identified using the rural-urban continuum codes from the US Department of Agriculture.

### Statistical Analysis

We performed multilevel logistic regression accounting for county-level clustering to estimate odds ratios (ORs) of surgery and radiotherapy. Models were adjusted for sociodemographic factors and cancer stage. Hazard ratios (HRs) of lung cancer–specific mortality were estimated using Fine and Gray subdistribution hazard models to treat death from non–lung cancer causes as a competing risk. We used robust sandwich covariate matrix estimates to account for intracounty dependence. Models were adjusted for sociodemographic factors and cancer stage and further for treatment. Trend was tested by including continuous geographic access scores in the models. We confirmed the proportional hazards assumption using the Schoenfeld residuals test. Missing values in covariates were included as a separate category. We stratified analyses by sociodemographic factors and evaluated their interactions with geographic access to thoracic surgeons and radiation oncologists. We conducted analyses using SAS, version 9.4 (SAS Institute Inc) from March to November 2024. Statistical significance was determined with a 2-sided *P* < .05.

## Results

Among 65 259 patients, the mean (SD) age was 69.4 (10.1) years; 33 114 (50.7%) were female and 32 145 (49.3%) were male; 3985 (6.1%) were Asian, 3239 (5.0%) were Hispanic, 6421 (9.8%) were non-Hispanic Black, 51 249 (78.5%) were non-Hispanic White, and 365 (0.6%) were of other race or ethnicity; 1071 (1.6%) were uninsured; 7541 (11.6%) had Medicaid; 11 666 (17.9%) were diagnosed with stage II disease; 13 045 (20.0%) lived in the most deprived counties; and 9224 (14.1%) lived in nonmetropolitan counties. Compared with patients in counties with the greatest access to thoracic surgeons, patients in counties with the least access were diagnosed at similar age and were more likely to be male, non-Hispanic White, Medicaid insured, living in the most deprived counties and nonmetropolitan counties, and diagnosed with stage II tumors (Table 1). Similar distributions of covariates were observed for patients in counties with the least (vs greatest) access to radiation oncologists.

**Table 1.** Characteristics of Patients With Early-Stage Non–Small Cell Lung Cancer in the SEER Dataset by Geographic Access to Thoracic Surgeons and Radiation Oncologists, 2007-2015

Characteristic	No. (%) of patients^a^						
	Total (N = 65 259^b^)	Geographic access to thoracic surgeons			Geographic access to radiation oncologists		
		Quintile 1 (least access) (n = 13 116)	Quintile 3 (n = 13 598)	Quintile 5 (greatest access) (n = 12 720)	Quintile 1 (least access) (n = 13 175)	Quintile 3 (n = 13 025)	Quintile 5 (greatest access) (n = 13 029)
Age, mean (SD), y	69.4 (10.1)	68.8 (9.8)	70.3 (10.0)	69.1 (10.3)	68.6 (9.8)	70.0 (10.1)	69.3 (10.2)
Age group, y							
20-49	1922 (3.0)	364 (2.8)	353 (2.6)	422 (3.3)	407 (3.1)	364 (2.8)	396 (3.0)
50-59	9003 (13.8)	1914 (14.8)	1628 (12.0)	1866 (14.7)	2005 (15.2)	1674 (12.9)	1845 (14.2)
60-69	20 820 (31.9)	4439 (33.8)	4169 (30.7)	4068 (32.0)	4477 (34.0)	4048 (31.1)	4155 (31.9)
70-79	22 555 (34.6)	4564 (34.8)	4866 (35.8)	4193 (33.0)	4522 (34.3)	4572 (35.1)	4396 (33.7)
≥80	10 959 (16.8)	1802 (13.7)	2582 (19.0)	2171 (17.1)	1764 (13.4)	2367 (18.2)	2237 (17.2)
Sex							
Male	32 145 (49.3)	6895 (52.6)	6593 (48.5)	6093 (47.9)	7051 (53.5)	6330 (48.6)	6189 (47.5)
Female	33 114 (50.7)	6221 (47.4)	7005 (51.5)	6627 (52.1)	6124 (46.5)	6695 (51.4)	6840 (52.5)
Race and ethnicity							
Asian	3985 (6.1)	282 (2.2)	1271 (9.4)	733 (5.8)	245 (1.9)	1279 (9.8)	816 (6.3)
Hispanic	3239 (5.0)	477 (3.6)	1196 (8.8)	422 (3.3)	553 (4.2)	917 (7.0)	433 (3.3)
Non-Hispanic Black	6421 (9.8)	989 (7.5)	1193 (8.8)	2179 (17.1)	1175 (8.9)	1166 (9.0)	1672 (12.8)
Non-Hispanic White	51 249 (78.5)	11 288 (86.1)	9857 (72.5)	9328 (73.3)	11 119 (84.4)	9592 (73.6)	10 021 (76.9)
Other^c^	365 (0.6)	80 (0.6)	81 (0.6)	58 (0.5)	83 (0.6)	71 (0.6)	87 (0.7)
Health insurance							
Non-Medicaid insurance	55 726 (85.4)	10 979 (83.7)	11 535 (84.8)	10 959 (86.2)	10 891 (82.7)	10 930 (83.9)	11 210 (86.0)
Medicaid	7541 (11.6)	1692 (12.9)	1769 (13.0)	1337 (10.5)	1853 (14.1)	1825 (14.0)	1431 (11.0)
No insurance	1071 (1.6)	256 (2.0)	168 (1.2)	235 (1.9)	261 (2.0)	152 (1.2)	201 (1.5)
Missing	921 (1.4)	189 (1.4)	126 (0.9)	189 (1.5)	170 (1.3)	118 (0.9)	187 (1.4)
County socioeconomic deprivation							
Quintile 1 (least)	13 947 (21.4)	931 (7.1)	2320 (17.1)	3451 (27.1)	1763 (13.4)	2998 (21.5)	2447 (18.8)
Quintile 5 (greatest)	13 045 (20.0)	5478 (41.8)	1212 (8.9)	2900 (22.8)	6131 (46.5)	1276 (9.8)	2922 (22.4)
Nonmetropolitan residence	9224 (14.1)	5914 (45.1)	738 (5.4)	534 (4.2)	5082 (38.6)	1163 (8.9)	596 (4.6)
Stage II cancer	11 666 (17.9)	2530 (19.3)	2306 (17.0)	2242 (17.6)	2521 (19.1)	2363 (18.1)	2211 (17.0)

Abbreviation: SEER, Surveillance, Epidemiology, and End Results.

^a^ Unless otherwise indicated.

^b^ Data were collected from the 17 SEER registries in 12 states, including 4 Western states (California, Hawaii, Washington, and Utah), 2 Northeastern states (Connecticut and New Jersey), 2 Midwestern states (Michigan and Iowa), and 4 Southern states (Georgia, Kentucky, Louisiana, and New Mexico).

^c^ Included 229 American Indian individuals, 63 individuals with another race, and 73 individuals with unknown race.

**Figure 1.**
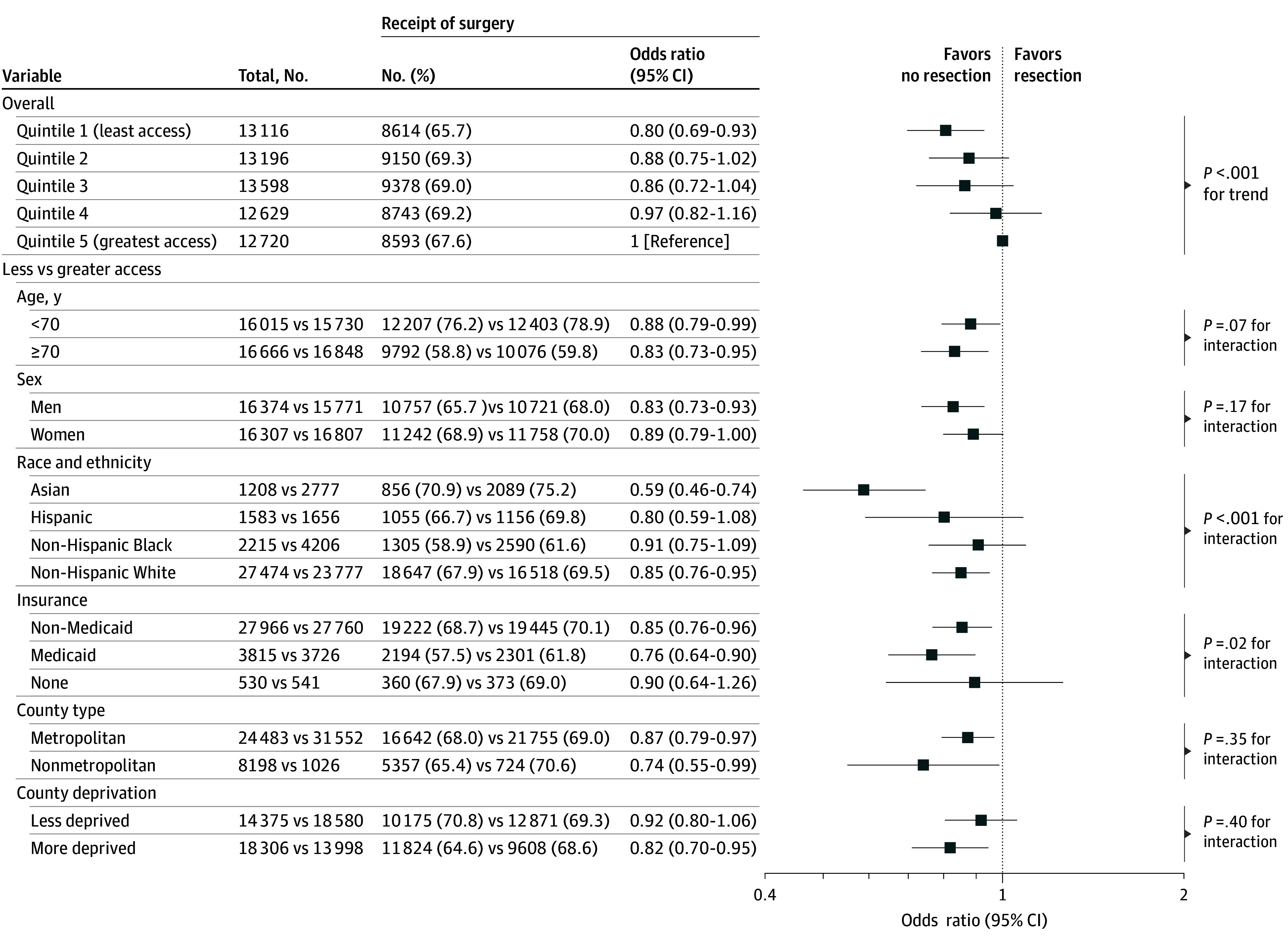
Association Between Geographic Access to Thoracic Surgeons and Surgery for Early-Stage Non–Small Cell Lung Cancer The models were adjusted for age, race and ethnicity, sex, type of health insurance, nonmetropolitan residence, quintiles of county-level socioeconomic deprivation index scores, cancer stage, and radiation therapy status. In the stratified analyses, less and greater geographic access was defined using the median of geographic access index scores. Less and more deprived counties were differentiated using the median of county-level socioeconomic deprivation index scores. The analysis stratified by race and ethnicity was restricted to Asian, Black, Hispanic, and White because there were too few cases in the other race and ethnicity group to obtain reliable estimates.

Overall, 41 795 patients (64.0%) underwent resection, 13 331 (20.4%) radiotherapy, 2583 (4.1%) both resection and radiotherapy, and 7450 (11.4%) neither surgery nor radiotherapy. After adjusting for sociodemographic factors, cancer stage, and radiotherapy, patients in counties with the least (vs greatest) access to thoracic surgeons were less likely to undergo resection (OR, 0.80; 95% CI, 0.69-0.93; *P* < .001 for trend). The association was much stronger in Asian patients (OR, 0.59; 95% CI, 0.46-0.74) than non-Hispanic White patients (OR, 0.85; 95% CI, 0.76-0.95) (*P* < .001 for interaction) and in Medicaid-insured patients (OR, 0.76; 95% CI, 0.64-0.90) than non–Medicaid-insured patients (OR, 0.85; 95% CI, 0.76-0.96) (*P* = .02 for interaction) (Figure 1).

**Figure 2.**
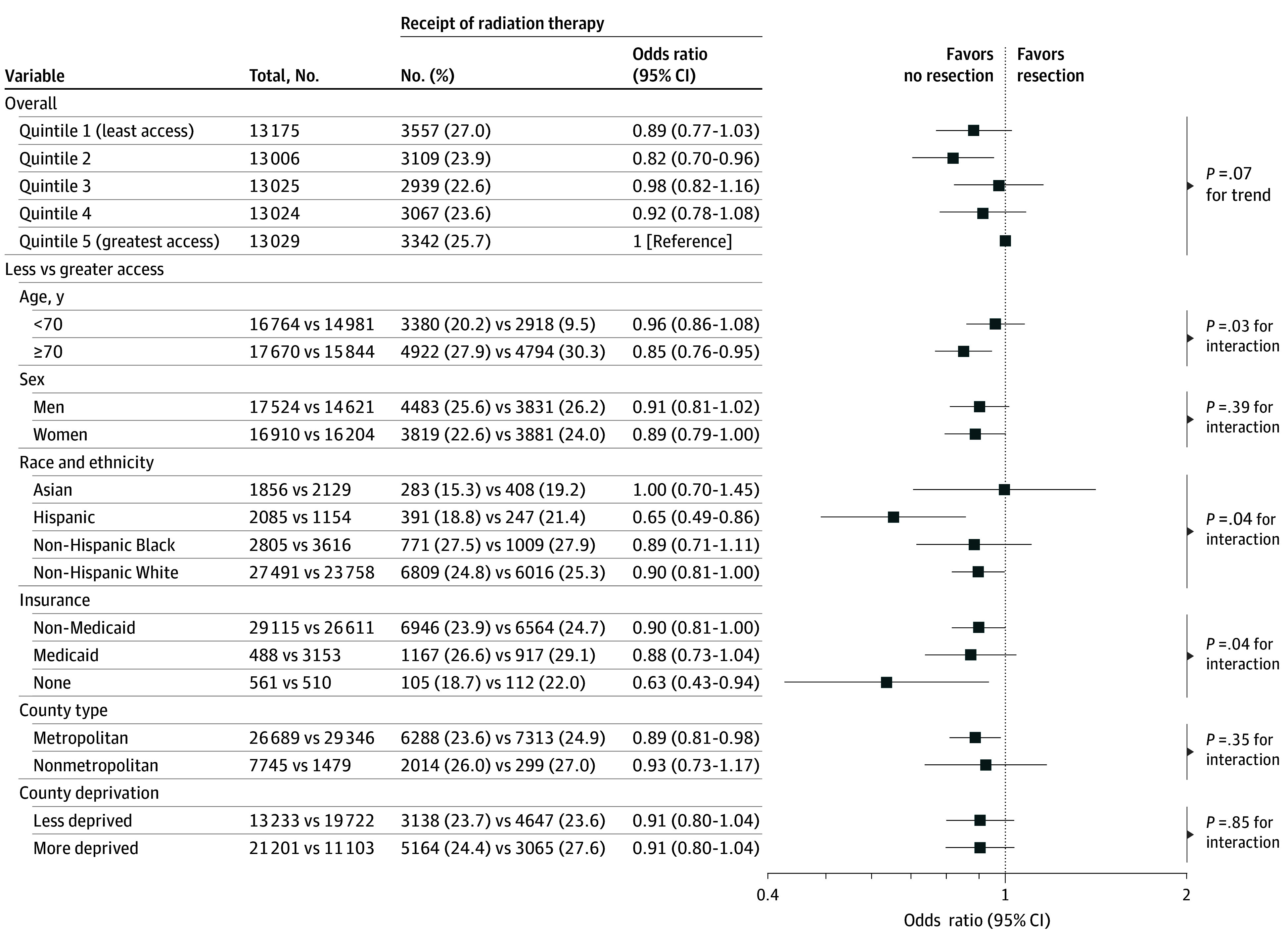
Association Between Geographic Access to Radiation Oncologists and Radiotherapy for Early-Stage Non–Small Cell Lung Cancer The models were adjusted for age, race and ethnicity, sex, type of health insurance, nonmetropolitan residence, quintiles of county-level socioeconomic deprivation index scores, cancer stage, and surgical treatment status. In the stratified analyses, less and greater geographic access was defined using the median of geographic access index scores. Less and more deprived counties were differentiated using the median of county-level socioeconomic deprivation index scores. The analysis stratified by race and ethnicity was restricted to Asian, Black, Hispanic, and White because there were too few cases in the other race and ethnicity group to obtain reliable estimates.

There was no significant association between geographic access to radiation oncologists and radiotherapy use (OR, 0.89; 95% CI, 0.77-1.03; *P* = .07 for trend) when adjusting for sociodemographic factors, cancer stage, and resection. However, the association significantly varied by age, race and ethnicity, and health insurance category. A significant association was observed in older (age ≥70 years) (OR; 0.85; 95% CI, 0.76-0.95), Hispanic (OR, 0.65; 95% CI, 0.49-0.86), and uninsured (OR, 0.63; 95% CI, 0.43-0.94) patients (Figure 2).

During a median (IQR) follow-up of 32 (15-60) months, 32 713 patients (50.1%) died and 22 149 (33.9%) died of lung cancer. Patients in counties with the least (vs greatest) access to thoracic surgeons (HR, 1.10; 95% CI, 1.03-1.18; *P* < .001 for trend) and those in counties with the least (vs greatest) access to radiation oncologists (HR, 1.11; 95% CI, 1.04-1.18; *P* < .001 for trend) had a higher subdistribution hazard of lung cancer mortality after adjusting for sociodemographic factors and cancer stage. Further adjustment for treatment reduced the HRs to 1.04 (95% CI, 0.97-1.12; *P* = .12 for trend) for access to thoracic surgeons and 1.07 (95% CI, 1.00-1.15; *P* = .01 for trend) for access to radiation oncologists (Table 2). A significant association with geographic access to thoracic surgeons was observed in more (HR, 1.10; 95% CI, 1.02-1.18) but not less (HR, 1.01; 95% CI, 0.96-1.06) deprived counties (*P* = .05 for interaction) (eTable 1 in Supplement 1). The association with geographic access to radiation oncologists was stronger in patients younger than 70 years (HR, 1.09; 95% CI, 1.03-1.15) than patients 70 years or older (HR, 1.06; 95% CI, 1.01-1.11) (*P* = .02 for interaction) (eTable 2 in Supplement 1).

**Table 2.** Associations Between Geographic Access to Cancer Specialty Care and Lung Cancer Mortality in Patients With Early-Stage Non–Small Cell Lung Cancer

Quintile	No. of person-years	No. of events	Adjusted HR (95% CI)^a^	Further adjusted HR (95% CI)^b^
**Geospatial accessibility to thoracic surgeons**				
Quintile 1 (least access)	41 751	4867	1.10 (1.03-1.18)	1.04 (0.97-1.12)
Quintile 2	43 284	4487	1.08 (1.01-1.15)	1.05 (0.99-1.12)
Quintile 3	44 487	4612	1.07 (1.00-1.15)	1.08 (1.00-1.16)
Quintile 4	43 082	3962	1.07 (1.00-1.14)	1.00 (0.93-1.08)
Quintile 5 (greatest access)	42 933	4221	1.00 [Reference]	1.00 [Reference]
*P* value for trend	NA	NA	<.001	.12
**Geospatial accessibility to radiation oncologists**				
Quintile 1 (least access)	41 064	4958	1.11 (1.04-1.18)	1.07 (1.00-1.15)
Quintile 2	42 376	4507	1.07 (1.01-1.13)	1.09 (1.03-1.15)
Quintile 3	42 963	4395	1.11 (1.05-1.17)	1.08 (1.01-1.16)
Quintile 4	44 447	4116	1.01 (0.95-1.07)	1.01 (0.95-1.07)
Quintile 5 (greatest access)	44 688	4173	1.00 [Reference]	1.00 [Reference]
*P* value for trend	NA	NA	<.001	.01

Abbreviations: HR, hazard ratio; NA, not applicable.

^a^ Adjusted for age, race and ethnicity, sex, type of health insurance, nonmetropolitan residence, quintiles of county-level socioeconomic deprivation, and cancer stage.

^b^ Further adjusted for cancer treatment.

## Discussion

In a nationally representative and geographically diverse cohort of patients with early-stage NSCLC, we observed that less geographic access to thoracic surgeons was associated with lower odds of undergoing resection and thereby a higher risk of lung cancer mortality. We also found that less geographic access to radiation oncologists was not associated with radiotherapy use but was associated with higher risk of lung cancer mortality. Of note, these associations were more pronounced in patients from socially marginalized populations, underscoring the importance of ensuring appropriate geographic allocation of cancer care resources and addressing travel barriers to health care to promote lung cancer health equity.

The National Comprehensive Cancer Network recommended that pulmonary resection should be performed by thoracic surgeons with a focus on lung cancer surgery.^5^ In a nationally representative cohort of patients undergoing lobectomy for lung cancer, 58.9% received surgery by thoracic surgeons, 30.1% by cardiovascular surgeons, and 11.0% by general surgeons.^27^ Surgeon specialty has been associated with surgical outcomes of patients with lung cancer, with better survival in patients treated by thoracic surgeons vs general surgeons.^28^ Although we could not distinguish resections by surgeon specialty, our results suggest that geographic access to thoracic surgeons may be a prognostic factor for patients with early-stage NSCLC by influencing whether they undergo resection.

We found that radiotherapy did not explain the association between geographic access to radiation oncologists and lung cancer mortality. Radiotherapy typically requires daily travel to treatment centers for several weeks. Therefore, completing a guideline-recommended course of radiotherapy may be more challenging for patients in neighborhoods with less access to radiation oncologists. Among patients with breast cancer, odds of radiotherapy interruption increased in those living more than 50 (vs <10) miles from the nearest radiotherapy facility.^29^ More research is needed to elucidate the association between geographic access to radiation oncologists and radiotherapy completion and its contribution to survival among patients with early-stage NSCLC.

Many older patients with lung cancer have medically inoperable disease, and radiotherapy is an appropriate treatment option for them.^30^ Our study showed that less geographic access to radiation oncologists was associated with lower odds of radiotherapy in patients 70 years or older but not patients younger than 70 years. Older patients often rely on family members and caregivers or public transportation for health care access.^31^ Therefore, transportation-related barriers, along with travel burden of radiotherapy, could explain lower likelihoods of radiotherapy for older patients in counties with less access to radiation oncologists. Interestingly, we found a stronger association between geographic access to radiation oncologists and mortality in younger patients. Considering that geographic access to radiation oncologists was not associated with radiotherapy use in younger patients, this result suggests that geographic access to radiation oncologists may play a critical role in radiotherapy completion in younger patients.

Studies have demonstrated that non-Hispanic Black patients were less likely than non-Hispanic White patients to receive surgery and radiotherapy for early-stage NSCLC.^1,32,33^ Our observation of no association of geographic access to thoracic surgeons or radiation oncologists with treatment in non-Hispanic Black patients suggests that geographic access to cancer care may not influence treatment decision and adherence in non-Hispanic Black patients with early-stage NSCLC. Lower likelihoods of receiving lung surgery from thoracic surgeons in non-Hispanic Black than non-Hispanic White patients^27^ could contribute to a null finding of geographic access to thoracic surgeons and surgery in this racial group. Additionally, research has found that some non-Hispanic Black patients had fatalistic views about lung cancer and misbelief toward lung cancer surgery and physician communication,^34,35^ which might partially explain lung surgery underuse in non-Hispanic Black patients.

In our study, Hispanic patients in counties with less access to radiation oncologists were less likely to receive radiotherapy. This finding could be explained by lack of transportation to health care facilities in Hispanic people^36^ and travel burden of radiotherapy. Among patients with early-stage NSCLC in the National Cancer Database, Hispanic and Asian patients were more likely than non-Hispanic White patients to undergo resection but less likely to receive radiotherapy.^1^ Therefore, our finding suggests that Hispanic patients with NSCLC may be more sensitive to geographic access to radiation oncologists compared with their non-Hispanic White counterparts, which may contribute to radiotherapy disparities among Hispanic patients.

In addition, we found that the association between geographic access to thoracic surgeons and undergoing resection was stronger in Asian than non-Hispanic White patients. This finding aligns with a study reporting lack of transportation and time as a common barrier to health care in Asian American populations.^37^ However, we did not see an association between geographic access to radiation oncologists and radiotherapy in Asian patients, suggesting that geographic access to radiation oncologists may not be an obstacle to radiotherapy for Asian patients. A qualitative study in Asian American communities reported that many Asian American people had inflexible working schedules or long-hour jobs.^37^ Given a requirement of repeated clinical encounters for radiotherapy, difficulties taking time off work might be a major obstacle to radiotherapy for Asian people.

Another key finding of our study is stronger associations between geographic access to lung cancer care and early-stage NSCLC treatment in uninsured and Medicaid-insured patients than non–Medicaid-insured patients. Only one study has specifically examined travel burden in relation to lung cancer care for Medicaid beneficiaries, which found that travel distance and time to a primary care physician was not associated with surgical therapy.^17^ Given a critical role of thoracic surgeons in the clinical management of lung cancer, we quantified geographic access to thoracic surgeons and found its association with undergoing resection of early-stage NSCLC in Medicaid enrollees. Alternatively, we observed an association between geographic access to radiation oncologists and radiotherapy in uninsured patients. Many uninsured patients gained Medicaid coverage shortly after their cancer diagnosis.^38^ Therefore, geographic access to thoracic surgeons and radiation oncologists may factor into Medicaid beneficiaries’ lung cancer treatment decisions. Nonemergency medical transportation services are available for Medicaid populations with no other transportation resources to clinical appointments. However, less than 4% of adult Medicaid beneficiaries used these services from 2018 to 2021, with use mainly for substance abuse treatment and preventive services.^39^ Despite the beneficial association of Medicaid expansion with early-stage diagnosis and survival in patients with NSCLC,^4^ Medicaid-insured patients still have suboptimal cancer treatment and worse survival compared with privately insured patients.^40^ With more patients with cancer gaining Medicaid coverage since Medicaid expansion,^41^ identifying barriers to high-quality cancer care for Medicaid beneficiaries is particularly important.

### Limitations

This study has some limitations. First, some results may be related to residual confounding from comorbidities and other unobserved factors. Second, SEER did not collect SBRT information. We considered receipt of external beam radiotherapy consistent with guidelines. For patients with inoperable disease who are unsuitable for SBRT, conventionally fractionated radiotherapy remains the standard of care.^42^ SEER might underascertain radiotherapy for lung cancer.^43^ Third, access to vehicles might modify the association between geographic access to cancer care and treatment use, which is the focus of our ongoing project. Fourth, county-level geographic access to cancer care might not accurately reflect access at smaller geographic units (eg, census tracts). Finally, the NPPES registry did not capture all thoracic surgeons and radiation oncologists in the US. More than 90% of radiation oncologists who are members of the American Medical Association were identified using NPPES.^44^

## Conclusions

In this cohort study, less geographic access to thoracic surgeons and radiation oncologists was associated with worse survival of patients with early-stage NSCLC. Importantly, geographic access to thoracic surgeons and radiation oncologists was more strongly associated with guideline-concordant treatment among patients 70 years or older, Hispanic patients, Asian patients, Medicaid enrollees, and uninsured patients, suggesting that these socially marginalized patients are sensitive to geographic barriers to cancer care. With introduction and implementation of low-dose computed tomography screening, more patients with lung cancer are expected to be diagnosed at early stages when tumors are highly treatable and often cured. Interventions targeting geographic barriers to lung cancer care could improve NSCLC treatment, prognosis, and equity.

## Notes

Supplement 1. **eTable 1.** Associations Between Geographic Access to Thoracic Surgeons and Lung Cancer-Specific Mortality Among Patients With Early-Stage Non-Small Cell Lung Cancer, Stratified by Sociodemographic Factors **eTable 2.** Associations Between Geographic Access to Radiation Oncologists and Lung Cancer-Specific Mortality Among Patients With Early-Stage Non-Small Cell Lung Cancer, Stratified by Sociodemographic Factors

Supplement 2.
Data Sharing Statement
