# Supplementary material for: Geographic Access to Cancer Care and Treatment and Outcomes of Early-Stage Non–Small Cell Lung Cancer
Source: JAMA Netw Open. 2025 Mar 18;8(3):e251061. doi: 10.1001/jamanetworkopen.2025.1061 (PMC11920842; doi:10.1001/jamanetworkopen.2025.1061)
Supplement: Supplement 1. — eTable 1. Associations Between Geographic Access to Thoracic Surgeons and Lung Cancer-Specific Mortality Among Patients With Early-Stage Non-Small Cell Lung Cancer, Stratified by Sociodemographic Factors eTable 2. Associations Between Geographic Access to Radiation Oncologists and Lung Cancer-Specific Mortality Among Patients With Early-Stage Non-Small Cell Lung Cancer, Stratified by Sociodemographic Factors [file jamanetwopen-e251061-s001.pdf]

Supplemental Online Content

Shrestha P, Liu Y, Struthers J, Kozower B, Lian M. Geographic access to cancer care and treatment and outcomes of early-stage non–small cell lung cancer. *JAMA Netw Open*. Published online March 18, 2025. doi:10.1001/jamanetworkopen.2025.1061

eTable 1. Associations Between Geographic Access to Thoracic Surgeons and Lung Cancer-Specific Mortality Among Patients With Early-Stage Non-Small Cell Lung Cancer, Stratified by Sociodemographic Factors

eTable 2. Associations Between Geographic Access to Radiation Oncologists and Lung Cancer-Specific Mortality Among Patients With Early-Stage Non-Small Cell Lung Cancer, Stratified by Sociodemographic Factors

This supplemental material has been provided by the authors to give readers additional information about their work.

© 2025 Shrestha P et al. *JAMA Network Open*.

eTable 1. Associations between geographic access to thoracic surgeons and lung cancer-specific mortality among patients with early-stage non-small cell lung cancer, stratified by sociodemographic factors.

	Events	Person-years	HR (95% CI)[c]
**Age <70 years**			
Less access[a]	4982	57804	1.03 (0.97, 1.08)
Greater access[a]	4430	59306	1.00 (reference)
**Age ≥70 years**			
Less access[a]	6586	47784	1.06 (1.01, 1.12)
Greater access[a]	6151	50645	1.00 (reference)
			$P_{interaction}$ =0.98
**Men**			
Less access[a]	6408	49534	1.03 (0.98, 1.08)
Greater access[a]	5721	49368	1.00 (reference)
**Women**			
Less access[a]	5160	56053	1.09 (1.02, 1.16)
Greater access[a]	4860	60583	1.00 (reference)
			$P_{interaction}$ =0.12
**Non-Hispanic white**			
Less access[a]	9759	89112	1.05 (1.01, 1.10)
Greater access[a]	7773	81051	1.00 (reference)
**Non-Hispanic black**			
Less access[a]	846	6715	1.10 (0.98, 1.22)
Greater access[a]	1504	13309	1.00 (reference)
**Asian**			
Less access[a]	352	4159	1.06 (0.86, 1.30)
Greater access[a]	766	9675	1.00 (reference)
**Hispanic**			
Less access[a]	559	5011	0.87 (0.72, 1.05)
Greater access[a]	516	5338	1.00 (reference)
			$P_{interaction}$ =0.52
**Non-Medicaid insurance**			
Less access[a]	9653	92119	1.06 (1.01, 1.11)
Greater access[a]	8833	95515	1.00 (reference)
**Medicaid**			
Less access[a]	1573	10564	1.06 (0.95, 1.19)
Greater access[a]	1356	10783	1.00 (reference)
**No insurance**			
Less access[a]	214	1745	0.98 (0.76, 1.26)
Greater access[a]	195	1766	1.00 (reference)
			$P_{interaction}$ =0.38
**Metropolitan**			
Less access[a]	8313	80305	1.06 (1.01, 1.11)
Greater access[a]	10207	106679	1.00 (reference)
**Non-metropolitan**			
Less access[a]	3255	25283	1.03 (0.92, 1.15)
Greater access[a]	374	3272	1.00 (reference)
			$P_{interaction}$ =0.75
**Less deprived counties[b]**			
Less access[a]	4506	48839	1.01 (0.96, 1.06)

© 2025 Shrestha P et al. *JAMA Network Open*.

Greater access[a]	5667	64565	1.00 (reference)
**More deprived counties[b]**			
Less access[a]	7062	56748	1.10 (1.02, 1.18)
Greater access[a]	4914	45386	1.00 (reference)
			$P_{interaction}$ =0.05

Abbreviations: HR, hazard ratio; CI, confidence interval.

[a] defined using the median of geographic access index scores.

[b] defined using the median of county-level socioeconomic deprivation index scores.

[c] adjusted for age, race and ethnicity, sex, type of health insurance, non-metropolitan residence, quintiles of county-level socioeconomic deprivation, and cancer stage.

© 2025 Shrestha P et al. *JAMA Network Open*.

eTable 2. Associations between geographic access to radiation oncologists and lung cancer-specific mortality among patients with early-stage non-small cell lung cancer, stratified by sociodemographic factors.

	Events	Person-years	HR (95% CI)[c]
**Age <70 years**			
Less access[a]	5306	59988	1.09 (1.03, 1.15)
Greater access[a]	4106	57121	1.00 (reference)
**Age ≥70 years**			
Less access[a]	6972	50504	1.06 (1.01, 1.11)
Greater access[a]	5765	47925	1.00 (reference)
			$P_{interaction}$ =0.02
**Men**			
Less access[a]	6885	52670	1.05 (1.00, 1.10)
Greater access[a]	5244	46233	1.00 (reference)
**Women**			
Less access[a]	5393	57823	1.12 (1.06, 1.18)
Greater access[a]	4627	58813	1.00 (reference)
			$P_{interaction}$ =0.17
**Non-Hispanic white**			
Less access[a]	9865	88635	1.08 (1.04, 1.13)
Greater access[a]	7667	81529	1.00 (reference)
**Non-Hispanic black**			
Less access[a]	1085	8507	1.05 (0.94, 1.17)
Greater access[a]	1265	11517	1.00 (reference)
**Asian**			
Less access[a]	548	6340	1.00 (0.86, 1.18)
Greater access[a]	570	7494	1.00 (reference)
**Hispanic**			
Less access[a]	734	6436	1.05 (0.89, 1.24)
Greater access[a]	341	3913	1.00 (reference)
			$P_{interaction}$ =0.71
**Non-Medicaid insurance**			
Less access[a]	10121	95551	1.07 (1.03, 1.12)
Greater access[a]	8365	92083	1.00 (reference)
**Medicaid**			
Less access[a]	1786	12022	1.12 (1.02, 1.23)
Greater access[a]	1143	9326	1.00 (reference)
**No insurance**			
Less access[a]	230	1808	1.06 (0.82, 1.36)
Greater access[a]	179	1703	1.00 (reference)
			$P_{interaction}$ =0.85
**Metropolitan**			
Less access[a]	9187	86742	1.07 (1.02, 1.12)
Greater access[a]	9333	100242	1.00 (reference)
**Non-metropolitan**			
Less access[a]	3091	23750	1.11 (1.03, 1.19)
Greater access[a]	538	4804	1.00 (reference)
			$P_{interaction}$ =0.41
**Less deprived counties[b]**			
Less access[a]	4267	44521	1.08 (1.03, 1.13)

© 2025 Shrestha P et al. *JAMA Network Open*.

Greater access[a]	5906	68884	1.00 (reference)
**More deprived counties[b]**			
Less access[a]	8011	65972	1.07 (1.00, 1.15)
Greater access[a]	3965	36162	1.00 (reference)
			$P_{interaction}$ =0.86

Abbreviations: HR, hazard ratio; CI, confidence interval.

[a] defined using the median of geospatial accessibility index scores.

[b] defined using the median of county-level socioeconomic deprivation index scores.

[c] adjusted for age, race and ethnicity, sex, type of health insurance, non-metropolitan residence, quintiles of county-level socioeconomic deprivation, and cancer stage.

© 2025 Shrestha P et al. *JAMA Network Open*.
