# Supplementary material for: Geographic Access to Cancer Care and Treatment and Outcomes of Early-Stage Non–Small Cell Lung Cancer
Source: JAMA Netw Open. 2025 Mar 18;8(3):e251061. doi: 10.1001/jamanetworkopen.2025.1061 (PMC11920842; doi:10.1001/jamanetworkopen.2025.1061)
Supplement: Supplement 2. — Data Sharing Statement [file jamanetwopen-e251061-s002.pdf]

Data Sharing Statement

Shrestha. Geographic Access to Cancer Care and Treatment and Outcomes of Early-Stage Non–Small Cell Lung Cancer. *JAMA Netw Open*. Published March 18, 2025.
doi:10.1001/jamanetworkopen.2025.1061

Data
Data available: No
